# Synthesis, Characterisation and In Vitro Anticancer Activity of Catalytically Active Indole-Based Half-Sandwich Complexes

**DOI:** 10.3390/molecules25194540

**Published:** 2020-10-03

**Authors:** Joan J. Soldevila-Barreda, Kehinde B. Fawibe, Maria Azmanova, Laia Rafols, Anaïs Pitto-Barry, Uche B. Eke, Nicolas P. E. Barry

**Affiliations:** 1 School of Chemistry and Biosciences, University of Bradford, Bradford BD1 7DP, UK; J.J.SoldevilaBarreda@bradford.ac.uk (J.J.S.-B.); M.Azmanova@bradford.ac.uk (M.A.); L.RafolsParellada@bradford.ac.uk (L.R.); a.pitto-barry@bradford.ac.uk (A.P.-B.); 2 Department of Chemistry, University of Ilorin, Ilorin P.M.B 1515, Nigeria; khennybee@gmail.com (K.B.F.); ekeub@yahoo.co.uk (U.B.E.)

**Keywords:** Indole, half-sandwich complexes, bioinorganic chemistry, catalytic drugs, anticancer

## Abstract

The synthesis, characterisation and evaluation of the in vitro cytotoxicity of four indole-based half-sandwich metal complexes towards two ovarian cancer cell lines (A2780 and A2780cisR) and one normal prostate cell line (PNT2) are presented herein. Although capable of inducing catalytic oxidation of NADH and able to reduce NAD^+^ with high turnover frequencies, in cells and in the presence of sodium formate, these complexes also strongly interact with biomolecules such as glutathione. This work highlights that efficient out-of-cells catalytic activity might lead to higher reactivity towards biomolecules, thus inhibiting the in-cells catalytic processes.

## 1. Introduction

Cancer is the second leading cause of death globally, with an estimated 9.6 million deaths in 2018 [1]. There is a need for the development and screening of anticancer therapeutics with non-conventional mechanisms of action (MoAs) [2,3,4]. Metallodrugs can provide rich chemistry and unique MoAs owing to their versatile structures, geometries and reactivities: examples include polypyridyl octahedral complexes of precious metals, some of which have been shown to target mitochondria and generate high levels of reactive oxygen species (ROS), while others can act as photosensitisers for use in photodynamic therapy [5,6,7,8,9,10,11], and half-sandwich complexes of metals from groups eight and nine (Fe, Ru, Os; Co, Rh, Ir) [12,13,14,15,16,17,18]. In this context, the binding of bioactive ligand(s) to metal fragments is of interest since this strategy may lead to the release of at least two biologically active species, therefore potentially offering enhanced activity against resistant cancer cells [19,20,21,22,23,24,25]. Ferrocene-quinoline conjugates (one of which completed phase II clinical trials) [23,26], antibacterial/antifungal quinolines attached to ruthenium organometallic fragments [20,22], or coordination of metal fragments to nonsteroidal anti-inflammatory drugs are some illustrations of such a strategy [21]. These results prompted us to study the effect of conjugating bio-active indole moieties to half-sandwich metal complexes.

Indoles are bicyclic heterocycles that are commonly found in plants, bacteria and animals. Natural and synthetic indole-based compounds have widely been used as antibacterial, antifungal, anti-inflammatory, antihistaminic and anticancer drugs [27,28]. Examples of such compounds currently in clinical use are the non-steroidal anti-inflammatory drug indomethacin [29] or the antiretroviral delavirdine [30]. On the anticancer front, numerous indole-based compounds have been developed to interfere with tubulin formation (necessary for cellular division) [31], inhibit histone deacilase [32,33] or sirtuins [34] (involved in gene expression), hamper DNA topoisomerase (involved in DNA transcription) or affect DNA directly via formation of inter- and intra-strand cross links [27,28]. Some notable examples are vincristine, a known antimitotic agent used for the treatment of some cancers [35,36], and mitomycin C, which undergoes in vivo reductive activation to form cross-links with DNA [37].

On a different note, in the last 10 years, much effort has been invested in studying the use of catalytic metal complexes for in-cells applications [38,39,40]. Particularly, the use of compounds capable of performing functional group modifications inside cells has attracted growing interest [38,39,40]. Such transformations not only allow the in-cells activation of pro-drugs or fluorophores through reduction [41,42] or deprotection of functional groups [43,44,45,46], but can also alter the homeostasis and metabolism of the cells via oxidation/reduction of molecules such as glutathione, nicotinamide dinucleotide or pyruvate/lactate [47,48,49,50,51,52]. Half-sandwich compounds of Ru(II), Os(II), Rh(III) or Ir(III) containing *N*,*N* chelating ligands have been specially successful at altering the NAD^+^/NADH ratio through transfer hydrogenation reactions [48,49,50,51,52,53,54,55]. The NAD^+^ and NADH pair have been shown to play crucial roles in many cellular metabolic processes, including the maintenance of redox homeostasis in the cell [56,57,58]. Alterations in oxidative stress have been shown to be particularly effective against cancer cells [47,50,52], which, owing to their active metabolism, are under constant oxidative stress [59].

**Figure 1:**
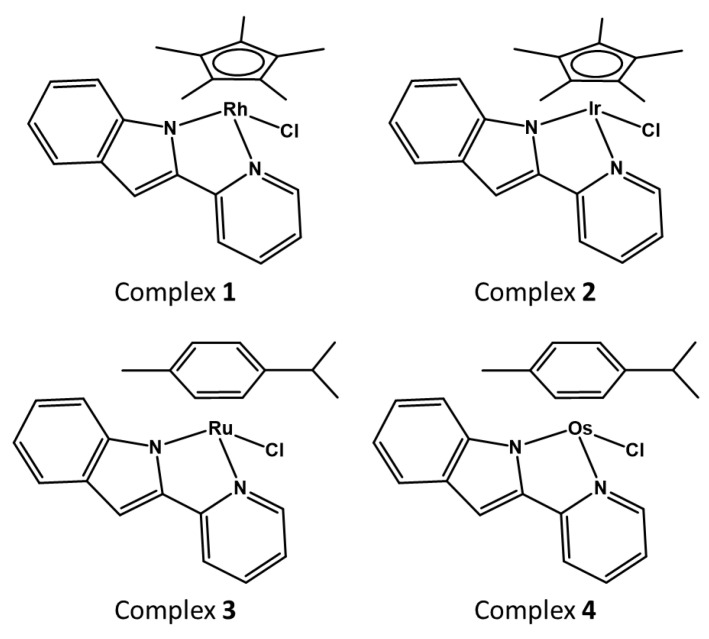
Molecular structures of complexes **1**–**4**.

Herein, we report the synthesis and characterisation of four half-sandwich complexes containing 2-(2-pyridinyl)-*1H*-indole (ind-py) ligands [Cp*Rh(ind-py)Cl] (**1**), [Cp*Ir(ind-py)Cl] (**2**), [(*p*-cym)Ru(ind-py)Cl] (**3**) and [(*p*-cym)Os(ind-py)Cl] (**4**) (Figure 1). The antiproliferative activity of these four organometallics towards ovarian cancer (A2780 and A2780cisR) and normal prostate (PNT2) cells is reported along with their stability in solution and reactivity with several potential (bio)ligands. Their catalytic activity towards reduction/oxidation of nicotinamide adenine dinucleotide coenzymes is also studied via NMR spectroscopy along with their in-cells catalytic activity via co-incubation with sodium formate and *N*-acetyl cysteine (NAC).

## 2. Results and Discussion

### 2.1. Synthesis, Stability in Solution, Aquation and pKa Determination

Complexes **1**–**4** were prepared by stirring corresponding metal dimers with the ligand 2-(2-pyridinyl)-*1H*-indole (ind-py) in dry dichloromethane at ambient temperature and in the presence of triethylamine. All complexes were characterised using ^1^H and ^13^C-NMR spectroscopy (Figures S2–S9), and high-resolution ESI-MS (Figures S10–S13).

Owing to the limited aqueous solubility of the complexes, all the reactivity experiments were performed using a mixture MeOD/D_2_O (1:1 *v*/*v*) as a solvent. The stability of complexes **1**–**4** in MeOD/D_2_O was studied via ^1^H-NMR spectroscopy over a period of 24 h. Under these conditions, only one set of resonances can be observed and no changes in the spectra occur over 24 h, which demonstrates that the complexes are fully stable under these conditions (Figure S1). The stability of complexes **1**–**4** in dimethylsulfoxide (DMSO;1.1 mM) was also studied by ^1^H-NMR spectroscopy over a period of 24 h (Figure S1). Complexes **1** and **2** were shown to release the ind-py ligand over time with a 25% decoordination rate over 24 h. Under biological testing conditions (medium/DMSO 99/1 *v*/*v*) it is expected that the release of ligand is greatly reduced. ^1^H-NMR spectra for complexes **3** and **4** show the formation of a second species, which is believed to be a DMSO-adduct of the complex. However, after reaching equilibrium in less than 10 min, no further changes can be observed, which demonstrates that the complexes are stable. The ligand ind-py is also stable in MeOD/D_2_O over 24 h (Figure S1E).

**Figure 2:**
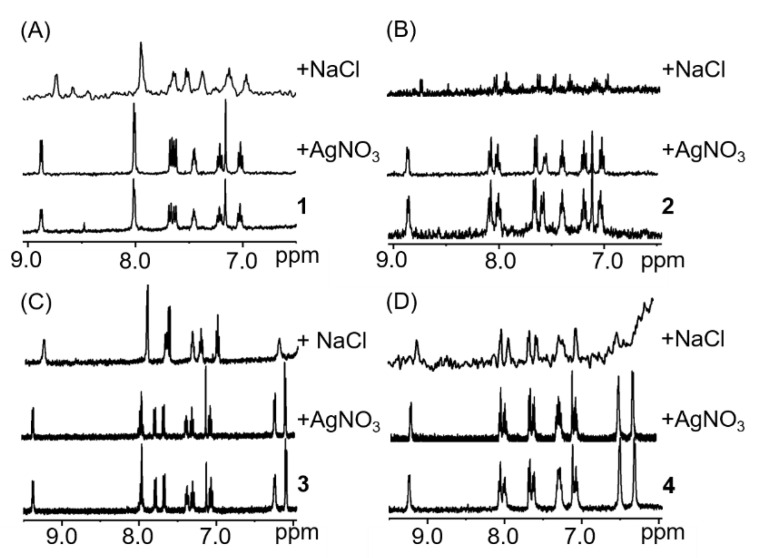
^1^H-NMR spectra of complexes **1**–**4** (**A**–**D**, respectively) in the presence of silver nitrate or sodium chloride, (1.1 mM complex, MeOD/D_2_O 1:1 *v*/*v*, pH 7.4, 298 K). Weak signals and low signal-to-noise ratios are due to the precipitation of the neutral chlorine complex adduct after addition of chlorine.

Aquation of the monodentate ligand (X) is a common behaviour for half-sandwich complexes of the type [(arene/Cp)M(*N*U*N*)X] and it is usually considered an activation step, which allows further reactions with the corresponding targets [39,60,61,62]. To study if aquation of complexes **1**–**4** occurs in MeOD/D_2_O (1:1 *v*/*v*), experiments in the presence of either one molar equivalent of silver nitrate (to force the release of the monodentate Cl^-^ ligand), or NaCl (to block hydrolysis), were performed. The addition of one molar equivalent of silver nitrate did not lead to any shift of the ^1^H-NMR signals compared to the ^1^H-NMR spectra of the complexes in MeOD/D_2_O in the absence of silver nitrate, which suggests that ligand substitution occurs immediately when adding MeOD/D_2_O even in the absence of silver ions, and the chloride ligand is replaced by either methanol or water (Figure 2). On the other hand, the addition of sodium chloride led to a different set of signals (Figure 2) and the concentrations of the species present in solution in the ^1^H-NMR samples were greatly reduced owing to major precipitation of the neutral chloride complexes.

The pKa values for the complexes were measured by dissolving the compounds in 6% acetonitrile/water *v*/*v* (aqua adduct formed) and by recording the UV-Vis spectra of the samples at increasing pH (pH 7–12). pKa values of the complexes were calculated using Origin 2018 by plotting the absorbance at the corresponding wavelength against the pH and fitting it to the Boltzmann equation to obtain the inflection point. For complexes **2**–**4**, the pKa values were calculated to be around 10 (Materials and Methods section and Table 1). The pKa of compound **1** could not be calculated, as the absorbance bands for the aqua- and the hydroxo- compounds overlap; however, changes could only be observed at pH values higher than 10. These pKa values are high, although such a decrease in acidity has been previously reported (generally attributed to an increased electron density on the metal centre) [60,63,64]. As a consequence of such high pKa, only the aqua adduct of the metal complexes is expected to be present at physiological pH (7.4), thus favouring the reaction between the metal complexes with possible ligands such as nucleobases or proteins.

### 2.2. Catalytic Reactions with Nicotinamide Adenine Dinucleotide

Some half-sandwich compounds containing ruthenium, osmium, rhodium or iridium metal centres have been shown to be able to perform transfer hydrogenation reactions in cells [38,39,41,47,65,66,67,68,69]. For example, iridium [(Cp^Xbiph^)Ir(phpy)x]^0/+^, osmium [(*p*-cym)Os(Azpy-NMe_2_)I]^+^ and rhodium [(Cp^X^)Rh(quin)X]^0/+^ (X = py or Cl) are capable of oxidising NADH to NAD^+^, thus affecting ROS homeostasis and numerous metabolic pathways [52,60,70,71].

**Figure 3:**
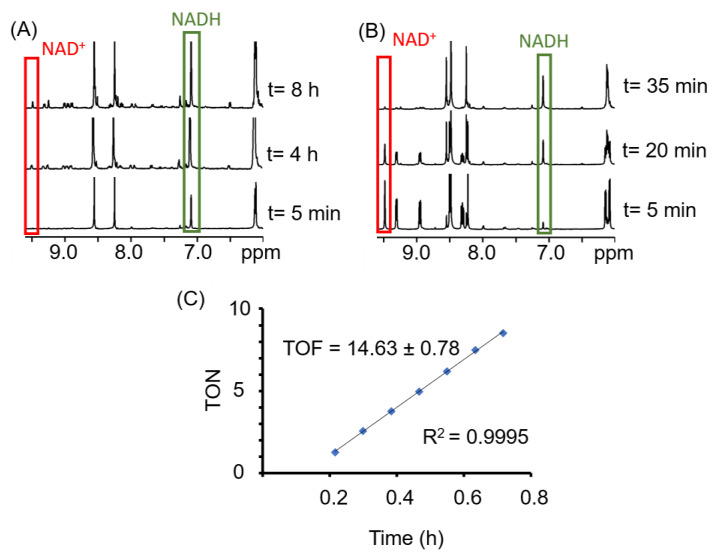
Kinetic experiments of complex **1** with 10 mol. equiv. NADH (**A**), or 9 mol. equiv. NAD^+^ and 28 mol. equiv. formate (**B**). Plot of the turnover number (TON) of the reaction of complex **1** with 28 mol. equiv. formate and 9 mol equiv. NAD^+^ versus time to calculate the turnover frequency (TOF) of the reaction (**C**). ^1^H-NMR spectra were recorded in MeOD/D_2_O (1/1 *v*/*v*), pH 7.4 and 310 K.

The ability of complexes **1**–**4** to oxidise NADH was investigated. The ^1^H-NMR spectra show an increase of the NAD^+^ signals over time (Figure 3). However, the reaction is very slow and after 8 h only around 10% of NADH is converted to NAD^+^. In the presence of hydride donors such as formate, many half-sandwich complexes of precious metals such as [(*p*-cym)Ru(TsEn)Cl] or [Cp*Rh(bip)Cl] have been shown to reduce different molecules, either in test tubes or even in cells [50,53,72,73]. The possibility of using compounds **1**–**4** as a transfer hydrogenation catalyst to reduce NAD^+^ was investigated (Materials and Methods section). Compounds **1**–**4** reduced NAD^+^ with turnover frequencies (TOF) between 6 and 15 h^−1^ (Table 1), which is within the range of previously reported compounds [51,53,74,75,76]. Remarkably, under the conditions here reported (MeOD/D_2_O 1:1 *v*/*v*, 310 K, pH* 7.4 ± 0.2), the iridium complex **2** reduced nine molar equivalents of NAD^+^ within the initial 10 min. However, no TOF could be calculated for this compound, owing to the speed of the reaction.

### 2.3. Chemosensitivity Assay

The antiproliferative activity of the ind-py ligand and of complexes **1**–**4** against ovarian cancer A2780 (cisplatin sensitive) and A2780cisR (cisplatin resistant) cell lines was studied. The IC_50_ values obtained for all the complexes are shown in Table 2, together with values for cisplatin, which was used as a positive control (untreated cells for 100% viability as negative control). The ruthenium compound **3** shows IC_50_ values up to 5–7× higher than cisplatin. Complexes **1**, **2** and **4** show IC_50_ values in the range of 20 µM, which is 3× less active than cisplatin itself. Interestingly, while complexes **1**–**3** are less active against A2780cisR than against A2780 cell lines, the osmium complex **4** shows no significant difference in activity, which suggests a MoA different from the one of cisplatin. One of the major limitations of existing anticancer drugs is their poor selectivity towards cancer cells. Comparing the response of the cancer cell lines to the normal PNT2 cells provides a preliminary indication towards the selectivity of the compounds. The IC_50_ values obtained for complexes **1**–**4** against PNT2 cells highlight that these compounds suffer from a lack of selectivity, although the Rh(III) and Os(II) compounds are 3× and 2× more selective towards cancerous cells (A2780) than towards normal cells (PNT2). The ind-py ligand was found to be non-cytotoxic against the three cell lines (Figure S14), which suggests that the activity of the complexes does not steam from ligand-release.

**Figure 4:**
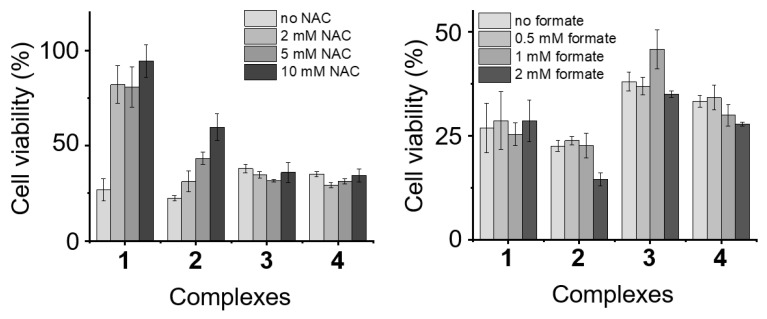
Cell viability of A2780 cells treated with complexes **1**–**4** in combination with NAC (Left) or sodium formate (Right).

Sadler et al. have recently demonstrated that oxidation of 1,4-NADH in cells could be a possible MoA to alter redox homeostasis of the cell, and therefore, could lead to cell death via an excess of ROS [52]. In order to determine whether ROS formation is a plausible explanation for the activity of these compounds, A2780 cells were treated at IC_50_ concentrations of **1**–**4** in combination with increasing concentrations of *N*-acetylcysteine (NAC; 0, 2, 5 and 10 mM; Figure 4). NAC is a known compound that can act as an ROS scavenger in cells and can be used to counteract the effects of complexes that affect ROS levels [76,77]. Interestingly, the addition of complexes **1** and **2** to NAC-supplemented cells leads to a significant decrease of the cell viability. This suggests that ROS generation might be involved in their MoAs. However, this effect is not observed with the Os and Ru analogues. Thus, although oxidation of 1,4-NADH could be involved in the MoA, it does not seem to be the main mechanism.

Encouraged by the catalytic properties of the complexes, we then investigated the possibility of increasing the antiproliferative activity of the compounds by treating the cells with a combination of complexes **1**–**4** (IC_50_ concentration) and sodium formate (0, 0.5, 1 and 2 mM). This approach has previously been reported with very promising results [50]. However, the treatment of A2780 and A2780cisR cells with IC_50_ concentrations of the complexes and increasing concentrations of sodium formate showed no significant effect. These results are not in agreement with our previous TOF calculations, which indicated that the complexes are capable catalysts for the reduction of NAD^+^. However, this lack of effect in cells is not completely unexpected. Do et al. have recently reported that some ruthenium compounds, although capable of performing transfer hydrogenation reactions inside cells using sodium formate as a hydride donor, did not induce enough change to destabilise the ROS homeostasis of the cell [68,69].

### 2.4. Reactions with Glutathione

**Figure 5:**
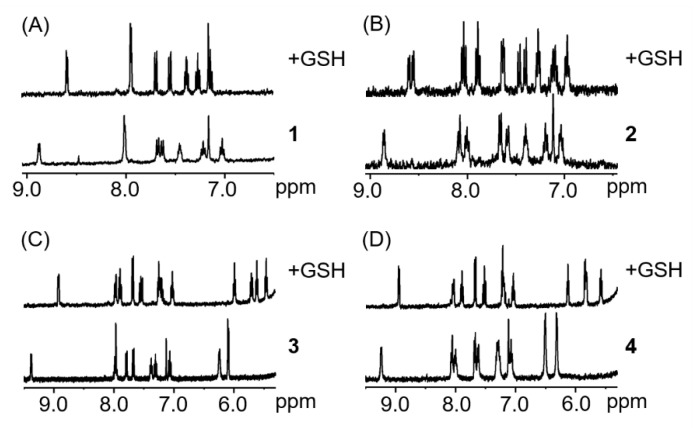
^1^H-NMR spectra of complexes **1**–**4** (**A**–**D**, respectively) in the presence of 2 mol. equiv. of GSH after less than 10 min (1.1 mM complex, MeOD/D_2_O 1:1 *v*/*v*, pH 7.4, 298 K).

To try to rationalise the discrepancy between the TOF calculations and the absence of effect in cells, we investigated the influence of glutathione (GSH) on the catalytic properties of complexes. GSH is a well-known detoxifying agent and ROS scavenger that is found in millimolar concentrations within the cell [78,79]. For example, it has been demonstrated that cisplatin-GSH adducts are easily formed, hampering the binding of cisplatin with DNA and contributing to the excretion of the drug from cells [80,81]. Similarly, half-sandwich complexes of ruthenium, osmium, iridium or rhodium have been reported to tightly bind to GSH, inhibiting any possible reaction with either DNA, NADH or other targets [82,83,84]. The interaction of complexes **1**–**4** with two equivalents of GSH was studied (1.1 mM complex, MeOD/D_2_O 1:1 *v*/*v*, pH 7.4, 298 K; Figure 5). ^1^H-NMR spectra of complexes **1**, **3** and **4** show only one set of signals, which can be attributed to the formation of the GSH adduct. Furthermore, the formation of glutathione adducts occurred within minutes, which demonstrates the high affinity of these complexes to bind to the tripeptide. Complex **2** was also shown to react within minutes after the addition of GSH, however two species can be observed in less than 10 min. Both species can be attributed to the reaction of the complex with GSH although the structure of the adduct could not be determined.

Complexes **1**–**4** promptly react with glutathione, yielding 100% of GSH adducts even in the presence of only one molar equivalent of glutathione. Thus, we decided to test the catalytic activity of the complexes towards the reduction of NAD^+^ in the presence of one molar equivalent of GSH. For this experiment, complex **2** was selected as a representative owing to its high catalytic activity compared to the other three complexes. After 2 h of reaction at 310 K, with one molar equivalent of GSH, nine molar equivalents of NAD^+^ and 28 molar equivalent of sodium formate, no formation of NADH can be observed. It is, therefore, unlikely that in the presence of millimolar concentrations of GSH, as well as many other potential binding molecules, the complexes will be able to perform as catalysts in cells.

### 2.5. Reactions with Nucleobases

**Figure 6:**
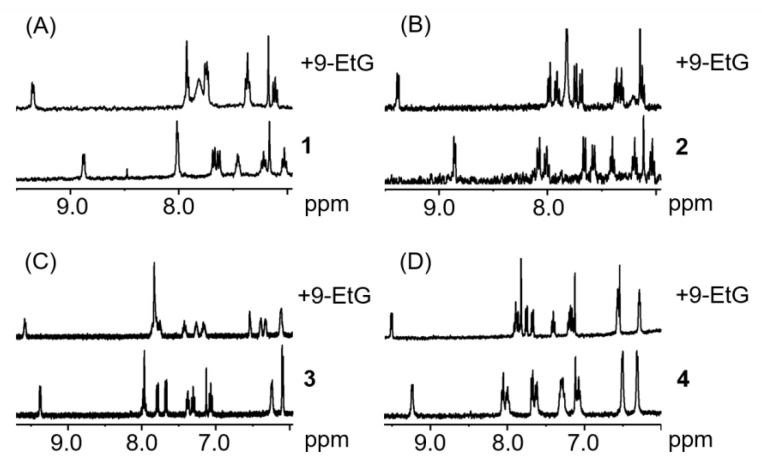
^1^H-NMR spectra of complexes **1**–**4** (**A**–**D**, respectively) in the presence of two mol. equiv. of 9-EtG after less than 10 min (1.1 mM complex, MeOD/D_2_O 1:1 *v*/*v*, pH 7.4, 298 K).

Since DNA is a known potential target for transition metals complexes [62,85], compounds **1**–**4** were reacted with two molar equivalents of 9-ethylguanine (9-EtG) and 9-methyladenine (9-MetA) in aqueous solution at pH 7.4 ± 0.2. All the complexes were shown to react with 9-EtG, with a full conversion within minutes. In MeOD/D_2_O (1:1 *v*/*v*), the H_8_ resonance of the 9-EtG (7.82 ppm under these conditions) can be seen to shift upfield by 1.3 ppm. Interestingly, from the ^1^H-NMR spectra of the complexes with 9-MetA (Figure 6), it can be observed that no reaction between the metal complexes and the nucleobase occurs. This demonstrates a more selective behaviour of the compounds hereby described compared to platinum drugs, which bind both to adenine and guanine [86].

Binding constant of complexes **1**, **3** and **4** with 9-EtG were calculated using the non-linear ThordarsonFittingProgram (Table 1) [87]. The binding constant for complex **2** was not calculated due to solubility issues. The magnitude of the binding constants (10^3^–10^4^ M^−1^) is low compared to the usually observed complexation constants in coordination chemistry (>>10^6^ M^−1^) [88], and is in the range of binding constants observed in host-guest inorganic chemistry (e.g., via non-covalent interactions between a metalla-cage and an aromatic planar guest molecule [89,90,91]). This suggests that these complexes could be weak DNA binders.

## 3. Conclusions

In conclusion, four half-sandwich metal complexes containing the bioactive indole moiety were synthesised and characterised. Their stability in solution was investigated and found sufficient to progress these compounds to in vitro screening. The complexes were found to be moderately active against A2780 ovarian cancer cells and A2780 cisplatin-resistant cell lines. Particularly, the Os(II) compound shows no significant difference in the IC_50_ against both cell lines, and therefore, no-cross resistance. Interestingly, the Rh(III) and Os(II) compounds show a 3× and 2× selectivity towards cancerous cells (A2780) versus normal cells (PNT2).

Catalytic oxidation of NADH using compounds **1**–**4** was demonstrated; however, the rate of the reaction is low, with only a 10% conversion after 12 h with most of the compounds. Furthermore, experiments in cells treated with the complexes and NAC as an ROS scavenger show that only the Ir(III) and Rh(III) might be generating oxidative stress, thus ruling out the NADH oxidation as a MoA. In contrast, all the complexes (**1**–**4**) were shown to reduce NAD^+^, in the presence of sodium formate, with turnover frequencies in the same high range as previously reported catalytic metallodrug candidates. Addition of glutathione to the catalytic reaction results in complete inhibition of NAD^+^ reduction. A2780 ovarian cancer cells treated with the complexes and sodium formate showed no increase on cytotoxic activity. These results demonstrate that complexes with high catalytic activity might correlate with higher reactivity towards other biomolecules, which concomitantly inhibits the catalytic process and in-cell activity.

## 4. Materials and Methods

### 4.1. Materials and Instrumentations

Roswell Park Memorial Institute (RPMI) 1640 medium, foetal bovine serum (FBS), penicillin and streptomycin, phosphate-buffered saline (PBS, pH 7.4) and other tissue culture reagents were purchased from Gibco (Thermo Fisher Scientific, UK). Non-dried solvents were purchased from Fischer Scientific and used as received. Dichloromethane was dried over molecular sieves. All the reactions were performed under standard Schlenk conditions unless otherwise specified. pH* was adjusted using EDT direction non-glass pocket pH meter with an ISFET silicon chip pH sensor. pH* values (pH readings without correction for the effect of deuterium) of NMR samples were adjusted using KOD solutions in D_2_O. All NMR spectra were recorded on a 400 MHz Bruker Spectrospin spectrometer using 5 mm NMR tubes. Data processing was carried out using TOPSPIN 3.5pl7 (Bruker U.K. Ltd.). Deuterated solvents were purchased from Goss Scientific Instrument. ^1^H-NMR chemical shifts were internally referenced to TMS via residual solvent peaks DMSO (*δ* = 2.52 ppm), CHCl_3_ (*δ* = 7.26 ppm), acetone (*δ* = 2.05 ppm), THF (*δ* = 1.72 ppm) or acetonitrile (*δ* = 1.94 ppm). All other chemicals were purchased from Sigma-Aldrich (UK). Cell lines were provided by the Institute of Cancer Therapeutics, University of Bradford, UK. Cells were incubated in a ThermoScientific HERAcell 150 incubator, and observed under a Nikon ECLIPSE TS100 Microscope.

### 4.2. Synthesis

[Cp*Rh(ind-py)Cl] (1): Rhodium dimer [(Cp*)RhCl_2_]_2_ (110 mg, 0.18 mmol) and 2-(2-pyridinyl)-1*H*-indole (ind-py, 78 mg, 0.38 mmol) were placed in a 50 mL 2-neck round-bottom flask and dissolved in 25 mL of dry dichloromethane. Dry triethylamine (66 µL, 0.47 mmol) was added to the reaction. The orange mixture was stirred under nitrogen overnight and at ambient temperature to obtain a dark orange solution. The reaction mixture was extracted with 0.1 M HCl solution (3 × 10 mL) and the combined organic layer was dried over magnesium sulphate, filtered and dried under vacuum to obtain an orange powder. The product was purified by chromatography column on silica gel with an acetone/dichloromethane (1:9 *v*/*v*) eluent and recrystallised in dichloromethane to obtain crystalline orange needles not suitable for X-ray. Yield: 63.4 mg (38.1%). HRMS-ESI^+^: calculated (M-Cl)^+^ 431.1000 *m*/*z*, found 431.0833 *m*/*z*. ^1^H NMR (400 MHz, CDCl_3_): δ_H_ 8.56 ppm (1H, d, *J* = 5.7 Hz, pyridyl-H-6), 7.74 (1H, d, *J* = 8.2 Hz, pyridyl-H-3), 7.68 (1H, t, *J* = 7.8 Hz, pyridyl-H-4), 7.61 (1H, d, *J* = 7.8 Hz, pyridyl-H-5), 7.46 (1H, d, *J* = 8.2 Hz, indole-H-4), 7.08 (2H, m, indole-H), 7.03 (1H, s, indole-H-3), 6.93 (1H, t, *J* = 7.6 Hz, indole-H), 1.66 (15H, s, CH_3_). ^13^C NMR (100 MHz, CDCl_3_): δ_C_ 150.2 ppm (pyridyl-C-6), 137.5 (pyridyl-C-4), 121.9 (indole-C-4 or -6), 121.2 (indole-C-4 or -6), 121.2 (pyridyl-C-5), 119.8 (pyridyl-C-3), 117.8 (indole-C-5), 115.3 (indole-C-7), 101.4 (indole-C-3), 9.5 (Cp).

[Cp*Ir(ind-py)Cl] (2): Complex **2** was synthesised following the preparation described for compound **1** with the following modifications: Ir dimer [(Cp*)IrCl_2_]_2_ (102 mg, 0.128 mmol), 2-(2-pyridinyl)-1*H*-Indole (50 mg, 0.257 mmol). The product was obtained as a yellow powder. Yield: 64.5 mg (27.2%). HRMS-ESI^+^: calculated (M-Cl)^+^ 521.1569 *m*/*z*, found 521.1564 *m*/*z*. ^1^H NMR (400 MHz, CDCl_3_): δ_H_ 8.55 ppm (1H, d, *J* = 5.6 Hz, pyridyl-H-6), 7.80 (1H, d, *J* = 8.3 Hz, pyridyl-H-3), 7.67 (1H, t, *J* = 7.8 Hz, pyridyl-H-4), 7.59 (1H, d, *J* = 7.8 Hz, pyridyl-H-5), 7.44 (1H, d, *J* = 8.5 Hz, indole-H-4), 7.05 (2H, m, 2 indole-H), 6.94 (2H, m, indole-H-3 and indole-H), 1.66 (15H, s, CH_3_). ^13^C NMR (100 MHz, CDCl_3_): δ_C_ 149.9 ppm (pyridyl-C-6), 137.5 (pyridyl-C-4), 121.9 (indole-C-4 or -6), 121.7 (indole-C-4 or -6), 121.3 (pyridyl-C-5), 119.3 (pyridyl-C-3), 117.9 (indole-C-5), 114.9 (indole-C-7), 102.2 (indole-C-3), 9.5 (Cp).

**[(*p*-cym)Ru(ind-py)Cl] (3):** Ruthenium dimer [(*p*-cym)RuCl_2_]_2_ (118.2 mg, 0.19 mmol) and 2-(2-pyridinyl)-1*H*-indole (75 mg, 0.38 mmol) were placed in a 50 mL 2-neck round-bottom flask and dissolved in 25 mL of dry dichloromethane. Dry triethylamine (54 μL, 0.38 mmol) was added to the reaction. The dark orange mixture was then left stirring under nitrogen overnight and at room temperature. The reaction mixture was extracted with 0.1 M HCl solution (3 × 10 mL) and the combined organic layer was dried over magnesium sulphate, filtered and dried under vacuum to obtain an orange powder. The product was purified by chromatography column on silica gel with an acetone/dichloromethane 10:90 *v*/*v* eluent. Yield: 72.1 mg (40.2%). HRMS-ESI^+^: calculated (M-Cl)^+^ 429.0905 *m*/*z*, found 429.0906 *m*/*z*. ^1^H NMR (400 MHz, CDCl_3_): δ_H_ 8.94 ppm (1H, d, *J* = 5.8 Hz, pyridyl-H-6), 7.73 (1H, d, *J* = 8.2 Hz, pyridyl-H-3), 7.68 (1H, d, *J* = 7.0 Hz, pyridyl-H-4), 7.63 (1H, d, *J* = 8.2 Hz, pyridyl-H-5), 7.52 (1H, d, *J* = 8.2 Hz, indole-H-4), 7.15 (2H, t, *J* = 7.5 Hz, 2 indole-H), 7.01 (3H, m, indole-H-3, indole-H, H_pcym_), 5.95 (1H, d, *J* = 6.1 Hz, H_pcym_), 5.59 (1H, d, *J* = 6.1 Hz, H_pcym_), 5.53 (1H, s, H_pcym_), 2.35 (1H, sept, *J* = 6.9 Hz, C*H*(CH_3_)_2_), 2.31 (3H, s, CH_3_), 0.88 (3H, d, *J* = 6.9 Hz, CH(C*H*_3_)_2_), 0.86 (3H, d, *J* = 6.9 Hz, CH(*CH*_3_)_2_). ^13^C NMR (100 MHz, CDCl_3_): δ_C_ 152.9 ppm (pyridyl-C-6), 137.3 (pyridyl-C-4), 121.9 (indole-C-4 or -6), 121.7 (indole-C-4 or -6), 120.8 (pyridyl-C-5), 119.8 (pyridyl-C-3), 117.9 (indole-C-5), 115.1 (indole-C-7), 101.1 (indole-C-3), 84.2 (CH_pcym_), 83.8 (CH_pcym_), 83.0 (CH_pcym_), 76.7 (CH_pcym_), 30.9 (CH_3_), 22.1 ((CH_3_)_2_), 22.0 ((CH_3_)_2_), 19.2 (CH).

**[(*p*-cym)Os(ind-py)Cl] (4):** Complex **4** was synthesised following the preparation described for compound **3** with the following modifications: Os dimer [(*p*-cym)OsCl_2_]_2_ (102 mg, 0.129 mmol), 2-(2-pyridinyl)-1*H*-indole (50 mg, 0.259 mmol). The product was obtained as a yellow powder. Yield: 74.8 mg (52.4%). HRMS-ESI^+^: calculated (M-Cl)^+^ 519.1476 *m*/*z*, found 519.1470 *m*/*z*. ^1^H NMR (400 MHz, CDCl_3_): δ_H_ 8.84 ppm (1H, d, *J* = 5.8 Hz, pyridyl-H-6), 7.81 (1H, d, *J* = 8.1 Hz, pyridyl-H-3), 7.68 (1H, m, pyridyl-H-4), 7.63 (1H, d, *J* = 8.0 Hz, pyridyl-H-5), 7.42 (1H, d, *J* = 8.3 Hz, indole-H-4), 7.13 (1H, dd, *J*_1_ = 8.3 Hz, *J*_2_ = 6.8 Hz, indole-H), 6.98 (2H, m, 2 indole-H), 6.95 (1H, s, indole-H-3), 6.28 (1H, d, *J* = 5.4 Hz, H_pcym_), 5.87 (1H, d, *J* = 5.4 Hz, H_pcym_), 5.80 (1H, d, *J* = 5.4 Hz, H_pcym_), 5.70 (1H, d, *J* = 5.4 Hz, H_pcym_), 2.42 (3H, s, CH_3_), 2.22 (1H, sept, *J* = 6.9 Hz, C*H*(CH_3_)_2_), 0.81 (3H, d, *J* = 6.9 Hz, (CH_3_)_2_), 0.79 (3H, d, *J* = 6.9 Hz (CH_3_)_2_). ^13^C NMR (100 MHz, CDCl_3_): δ_C_ 152.8 ppm (pyridyl-C-6), 137.5 (pyridyl-C-4), 122.0 (indole-C-4 or -6), 121.8 (indole-C-4 or -6), 121.3 (pyridyl-C-5), 119.5 (pyridyl-C-3), 118.2 (indole-C-5), 115.2 (indole-C-7), 101.6 (indole-C-3), 77.2 (CH_pcym_), 74.7 (CH_pcym_), 72.6 (CH_pcym_), 66.4 (CH_pcym_), 31.3 (CH_3_), 22.5 ((CH_3_)_2_), 22.4 ((CH_3_)_2_), 19.1 (CH).

### 4.3. Solution Chemistry

Complexes **1**–**4** were dissolved in MeOD (2.2 mM) and diluted to a final concentration of 1.1 mM with either MeOD or D_2_O. ^1^H-NMR spectra were recorded at *t* ≤ 10 min, 12 h and 24 h. The samples were then reacted with either 1 mol. equiv. of silver nitrate to obtain the fully hydrolysed species or NaCl to block hydrolysis.

Complexes **1**–**4** were also dissolved in d_6_-DMSO (1.1 mM) and ^1^H-NMR spectra were recorded at *t* ≤ 10 min, 12 h and 24 h.

Complexes **1**–**4** were dissolved in acetonitrile/H_2_O 6:94 *v*/*v* and UV-Vis spectra of the sample were recorded increasing the pH (pH 7–12). pKa vales of the complexes were calculated using Origin 2018 by plotting the absorbance against the pH.

### 4.4. Reaction with Nucleobases

The reactions of complexes **1**–**4** with 9-ethylguanine (9-EtG) and 9-methyladenine (9-MetA) were studied. MeOD solutions of complexes **1**–**4** (2.2 mM) were mixed with a solution of the corresponding nucleobase in D_2_O (4.4 mM) and the pH adjusted to 7.4 ± 0.2. Final concentrations of 1.1 mM for the complex and 2.2 mM for the nucleobase were obtained. ^1^H-NMR spectra of the resulting mixture were recorded at t ≤ 10 min, 5 h and 24 h in MeOD/D_2_O (1:1 *v*/*v*).

A titration using 9-EtG at increasing concentrations was performed and the resulting samples were analysed using ^1^H-NMR spectroscopy. Binding constants were calculated using the non-linear ThordarsonFittingProgram [87] by plotting the chemical shift of the complex versus the concentration of 9-EtG. Alternatively, binding constant for complexes **3** and **4** was obtained by plotting the ratio of 9-EtG adduct/complex against the concentration of 9-EtG.

### 4.5. Reaction with Glutathione

The reactions of complexes **1**–**4** with glutathione (GSH) were studied. MeOD solutions of complexes **1**–**4** (2.2 mM) were mixed with a solution of GSH in D_2_O (10 mM) and the pH was adjusted to 7.4 ± 0.2. Final concentrations of 1.1 mM for the complex, and 2.2 mM for GSH were obtained. ^1^H-NMR spectra of the resulting mixture were recorded at *t* ≤ 10 min, 5 h and 24 h in MeOD/D_2_O (1:1 *v*/*v*).

### 4.6. Oxidation of 1,4-NADH

The interactions of complexes **1**–**4** with 1,4-NADH were studied by mixing a solution of the corresponding complex (2.2 mM, MeOD) and 1,4-NADH (22 mM, D_2_O). The pH of the solution was then adjusted to 7.4 ± 0.2 and ^1^H-NMR spectra recorded at 310 K at various time intervals. Final concentrations of 1.1 mM for the complex and 10 mM for 1,4-NADH were obtained. ^1^H-NMR spectra of the resulting mixture were recorded in MeOD/D_2_O (1:1 *v*/*v*).

### 4.7. NAD^+^ Reduction

Transfer hydrogenation reactions with complexes **1**–**4**, formate, and NAD^+^ were studied by mixing a solution of the corresponding complex (2.2 mM, MeOD), sodium formate (137 mM, D_2_O) and NAD^+^ (44 mM, D_2_O). Final concentrations of 1.1 mM for the complex, 10 mM for NAD^+^ and 30 mM for NaHCO_2_ were obtained (molar ratio 1:9:28). The pH of the mixture was adjusted to 7.4 ± 0.2. ^1^H-NMR spectra were recorded at 310 K every 5 min until completion of the reaction.

Molar ratios of NAD^+^ and NADH were determined by integrating the area under the signals associated with NAD^+^ (9.33 ppm) and 1,4-NADH (6.96 ppm). The turnover number (TON) for the reaction was calculated as follows:

(1) $$\mathrm{TON}\frac{I_{6.96}}{I_{6.96}+I_{9.93}}\frac{{\left[{\mathrm{NAD}}^{+}\right]}_{0}}{\left[\mathrm{Catalyst}\right]}$$

where I*_n_* is the integral of the signal at *n* ppm and [NAD^+^]_0_ is the concentration of NAD^+^ at the start of the reaction.

### 4.8. Inhibition of NAD^+^ Reduction by Glutathione

Following the experimental procedure for the reduction of NAD^+^, a solution of complex **2** was mixed with NAD^+^, NaHCO_2_ and GSH and the pH of the resulting solution adjusted to 7.4 ± 0.2. Final concentrations were as follows: complex 1.1 mM; NAD^+^ 10 mM; NaHCO_2_ 30 mM; GSH 1.1 mM; molar ratio 1:9:28:1. ^1^H-NMR spectra were recorded at 310 K every 162 s for a period of 2 h.

### 4.9. Chemosensitivity Assays

In vitro chemosensitivity tests were performed against A2780, A2780cisR and PNT2 cells. Cells were routinely maintained as monolayer cultures in RPMI medium supplemented with 10% foetal calf serum, penicillin (100 I.U./mL) and streptomycin (100 μg/mL), sodium pyruvate (1 mM) and L-glutamine (2 mM). For chemosensitivity studies, cells were incubated in 96-well plates at a concentration of 7.5 × 10^3^ cells per well and the plates were incubated for 24 h at 37 °C and a 5% CO_2_ humidified atmosphere prior to drug exposure.

Complexes were dissolved in DMSO to provide stock solutions, which were further diluted with media to provide a range of final concentrations. Drug-media solutions were added to cells (the final concentration of DMSO was less than 1% (*v*/*v*) in all cases) and incubated for 24 h at 37 °C and 5% CO_2_ humidified atmosphere. The drug-media was removed from the wells and the cells were washed with PBS (100 μL, twice), and 100 μL of complete fresh media were added to each well. The plates were further incubated for 48 h at 37 °C in a 5% CO_2_ humidified atmosphere to allow for a period of recovery. 3-(4,5-dimethylthiazol-2-yl)-2,5-diphenyltetrazolium bromide (MTT) (20 μL, 2.5 mg/mL) was added to each well and incubated for 2 h at 37 °C and 5% CO_2_ humidified atmosphere. All solutions were then removed and 100 μL of DMSO was added to each well in order to dissolve the purple formazan crystals. A Thermo Scientific Multiskan EX microplate photometer was used to measure the absorbance in each well at 570 nm. Cell survival was determined as the absorbance of treated cells divided by the absorbance of controls and expressed as a percentage. The IC_50_ values were determined from plots of % survival against drug concentration. Each experiment was repeated in triplicates of triplicates and a mean value was obtained and stated as IC_50_ (μM) ± SD. Cisplatin was also used as a positive control.

### 4.10. Cell Viability Experiments

Cells were incubated in 96-well plates at a concentration of 7.5 × 10^3^ cells per well and the plates were incubated for 24 h at 37 °C and a 5% CO_2_ humidified atmosphere prior to drug exposure. Complexes were dissolved in DMSO to provide stock solutions, which were further diluted with media to working concentrations. Solution of *N*-acetylcysteine (NAC) or sodium formate in media were also prepared. Drug-media solutions and NAC or sodium formate solutions were added to cells (final concentrations as follows: IC_50_ values for the complex; 0, 2, 5 and 10 mM for NAC; 0, 0.5, 1 and 2 mM for sodium formate; DMSO concentration was less than 1% (*v*/*v*) in all cases) and incubated for 24 h at 37 °C in a 5% CO_2_ humidified atmosphere. After 24 h of incubation, the drug-media was removed from the wells and the cells were washed with PBS (100 μL, twice), and 100 μL of complete fresh media were added to each well. The plates were further incubated for 48 h at 37 °C in a 5% CO_2_ humidified atmosphere to allow for a period of recovery. 3-(4,5-dimethylthiazol-2-yl)-2,5-diphenyltetrazolium bromide (MTT) (20 μL, 2.5 mg/mL) was added to each well and incubated for 2 h at 37 °C and 5% CO_2_ humidified atmosphere. All solutions were then removed and 100 μL of DMSO was added to each well in order to dissolve the purple formazan crystals. A Thermo Scientific Multiskan EX microplate photometer was used to measure the absorbance in each well at 570 nm. Cell survival was determined as the absorbance of treated cells divided by the absorbance of controls and expressed as a percentage.

## Figures and Tables

**Table 1:** Reactivity of complexes **1**–**4**. ^a^ pKa values measured using UV-Vis spectroscopy, solvent 6% acetonitrile/water *v*/*v*. ^b^ Binding constants towards 9-EtG, concentration of complex 1.1 mM in MeOD/D_2_O (1:1 *v*/*v*). ^c^ Turnover frequencies for the reduction of NAD^+^ (9 mol. equiv.) using formate (28 mol. equiv.) as a hydride donor, pH* 7.4 ± 0.2, 310 K. Concentration of complex 1.1 mM in MeOD/D_2_O (1:1 *v*/*v*).

Compound	pKa ^a^	*K*_9-EtG_ (M^−1^) ^b^	TOF (h^−1^) ^c^
[Cp*Rh(ind-py)Cl] (**1**)	-	25.6 × 10^3^	14.6 ± 0.7
[Cp*Ir(ind-py)Cl] (**2**)	9.59 ± 0.09 (λ = 300 nm)	-	-
[(*p*-cym)Ru(ind-py)Cl] (**3**)	10.63 ± 0.03 (λ = 255 nm)	71.6 × 10^3^	7.95 ± 0.81
[(*p*-cym)Os(ind-py)Cl] (**4**)	10.15 ± 0.04 (λ = 355 nm)	95.4 × 10^3^	5.74 ± 0.35

**Table 2:** IC_50_ values (µM) in A2780, A2780 cisplatin resistant and PNT2 cells for complexes **1**–**4**.

	IC_50_ Values (µM)		
Compound	A2780	A2780cisR	PNT2
[Cp*Rh(ind-py)Cl] (**1**)	13.0 ± 1.7	21.5 ± 3.5	34.7 ± 6.1
[Cp*Ir(ind-py)Cl] (**2**)	22.0 ± 2.5	32.7 ± 1.7	32.1 ± 3.9
[(*p*-cym)Ru(ind-py)Cl] (**3**)	47.3 ± 4.7	62.8 ± 5.3	54.3 ± 5.4
[(*p*-cym Os)(ind-py)Cl] (**4**)	18.8 ± 0.3	21.5 ± 1.3	34.2 ± 2.2
Ind-py	>100	>100	>100
Cisplatin	10.3 ± 0.5	22.4 ± 0.5	43 ± 3

## Supplementary Materials

The following are available online, Figure S1: ^1^H NMR stability studies; Figures S2–S9: ^1^H and ^13^C NMR spectra of complexes **1**–**4**, respectively; Figures S10–S13: High-resolution ESI mass spectrum of complexes **1**–**4**, respectively; Figure S14: IC_50_ graphs for the ind-py ligand against PNT2, A2780, and A2780cisR.
