# Supplementary material for: Synthesis, Characterisation and In Vitro Anticancer Activity of Catalytically Active Indole-Based Half-Sandwich Complexes
Source: Molecules. 2020 Oct 3;25(19):4540. doi: 10.3390/molecules25194540 (PMC7583056; doi:10.3390/molecules25194540)
Supplement: Supplementary file 1 [file molecules-25-04540-s001.pdf]

Electronic Supplementary Information

Synthesis, characterisation, and *in vitro* anticancer activity of catalytically active indole-based half-sandwich complexes.

Joan J. Soldevila-Barreda[1], Kehinde B. Fawibe[2], Maria Azmanova[1], Laia Rafols[1], Anaïs Pitto-Barry[1], Uche B. Eke[2] and Nicolas P. E. Barry[1,*]

[1] School of Chemistry and Biosciences, University of Bradford, BD1 7DP, Bradford, United Kingdom
[2] Department of Chemistry, University of Ilorin, P.M.B 1515, Ilorin, Nigeria
* Correspondence: N.Barry@Bradford.ac.uk

Figure S1: ^{1}H NMR stability studies

Figures S2 – S9: ^{1}H and ^{13}C NMR spectra of complexes **1 – 4**

Figures S10 – S13: High-resolution ESI mass spectrum of complexes **1 – 4**

Figure S14: IC_{50} graphs for the ind-py ligand against PNT2, A2780, and A2780cisR.

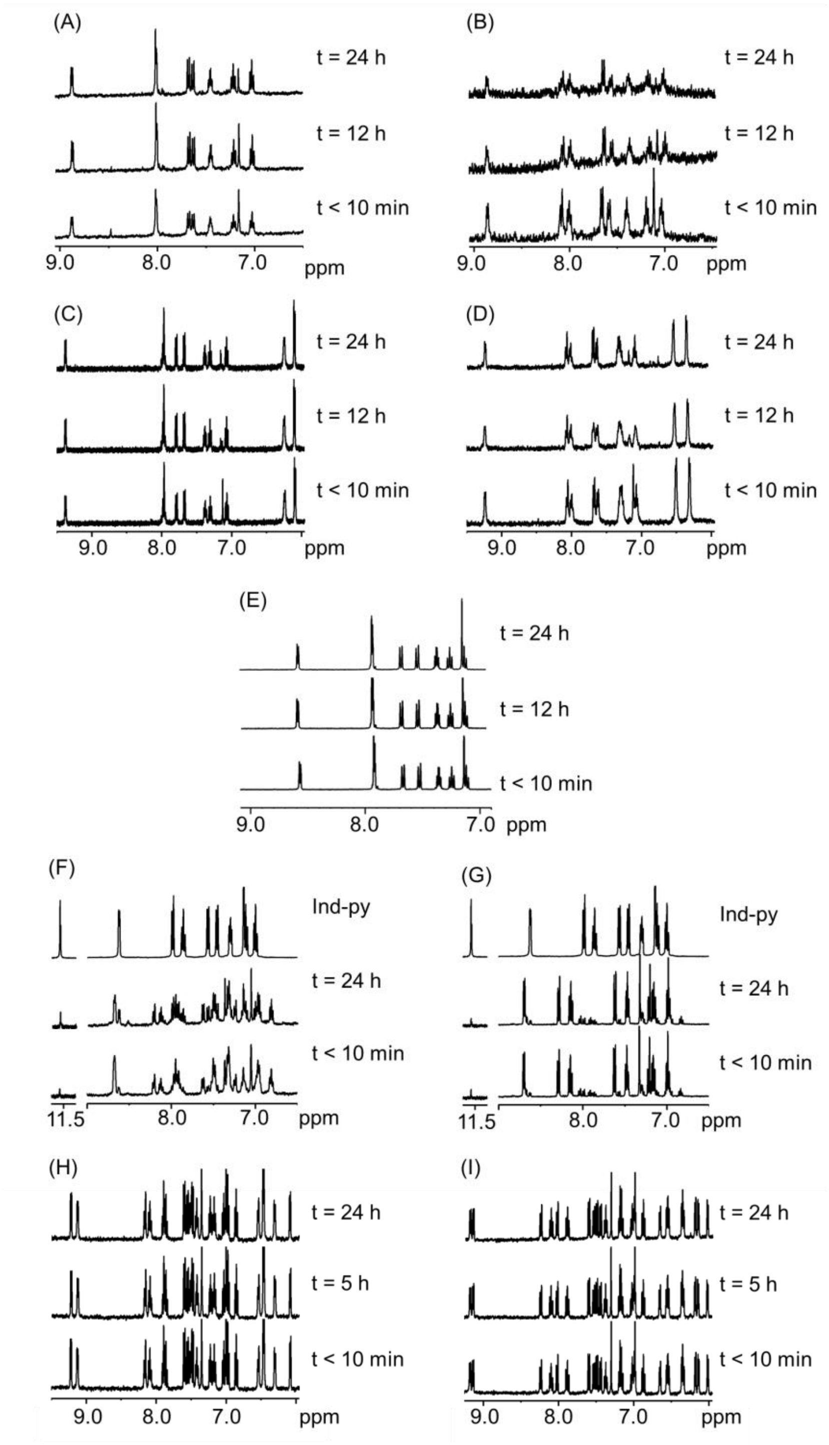

Figure 1. ^{1}H NMR (400 MHz) stability studies of complex **1** (A), **2** (B), **3** (C), **4** (D), and Ind-Py (E) in MeOD/D_2O (1:1 v/v, 1.1 mM, 298 K) and complexes **1** (E), **2** (F), **3** (G) and **4** (H) in d_6-DMSO (1.1 mM, 298 K). The low signal-to-noise ratio for (B) is due to the poor solubility of complex **2** in MeOD/D_2O.

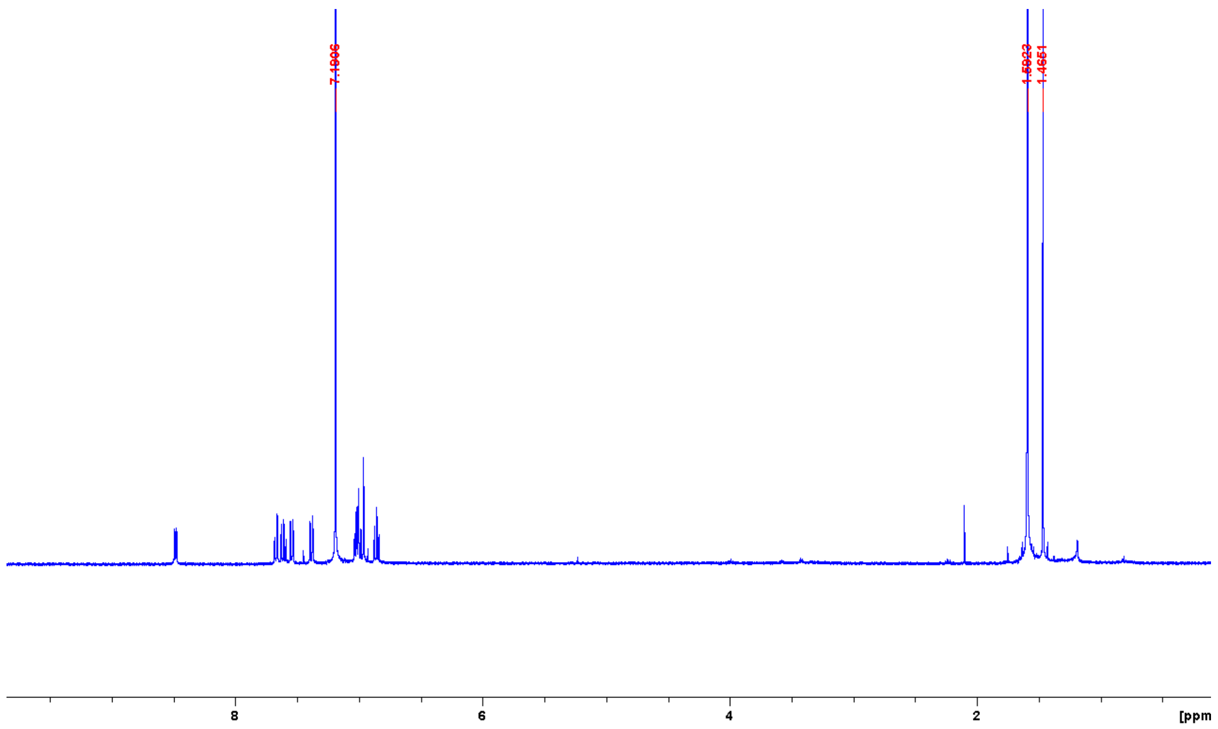

Figure S2. ^{1}H NMR spectrum of complex **1**

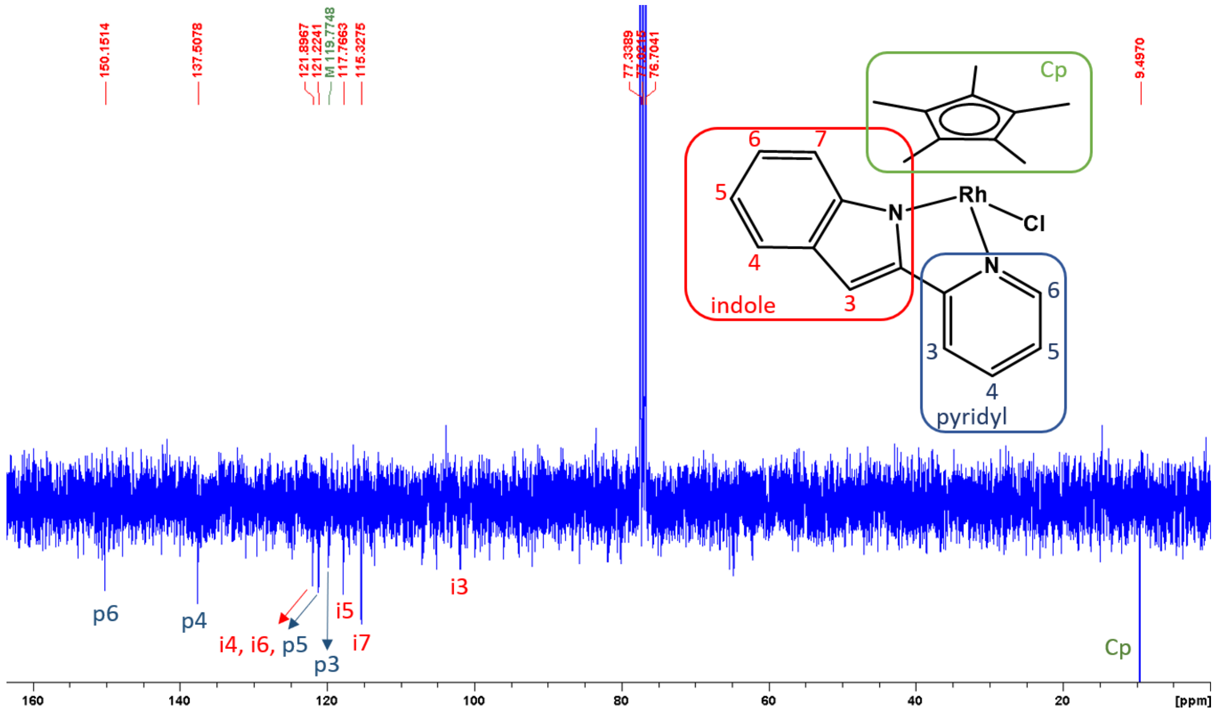

Figure S3. ^{13}C NMR spectrum of complex **1**

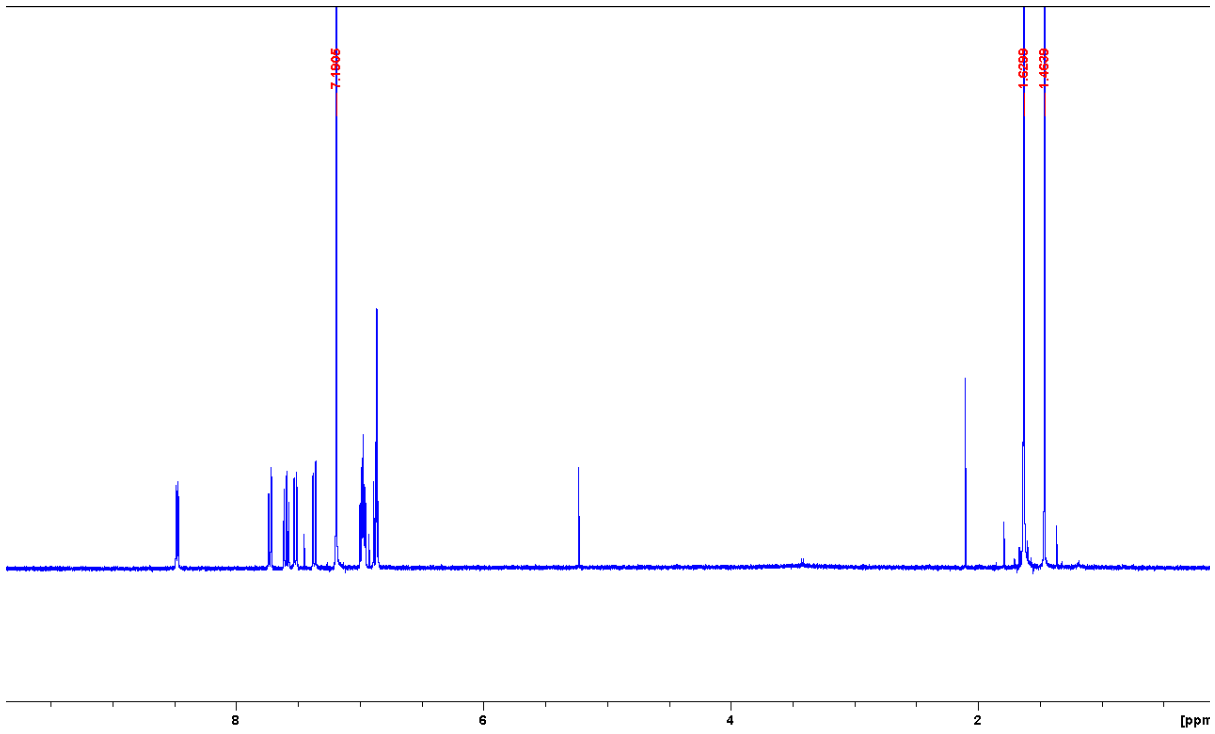

Figure S4. ^{1}H NMR spectrum of complex **2**

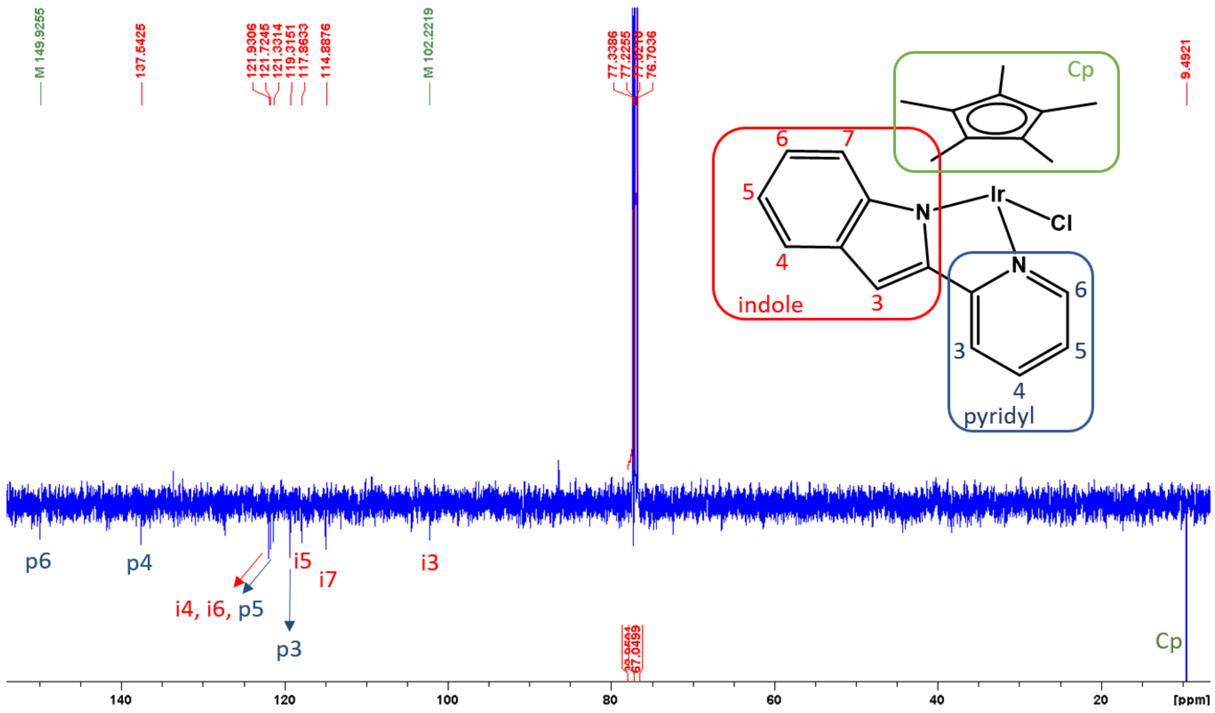

Figure S5. ^{13}C NMR spectrum of complex **2**

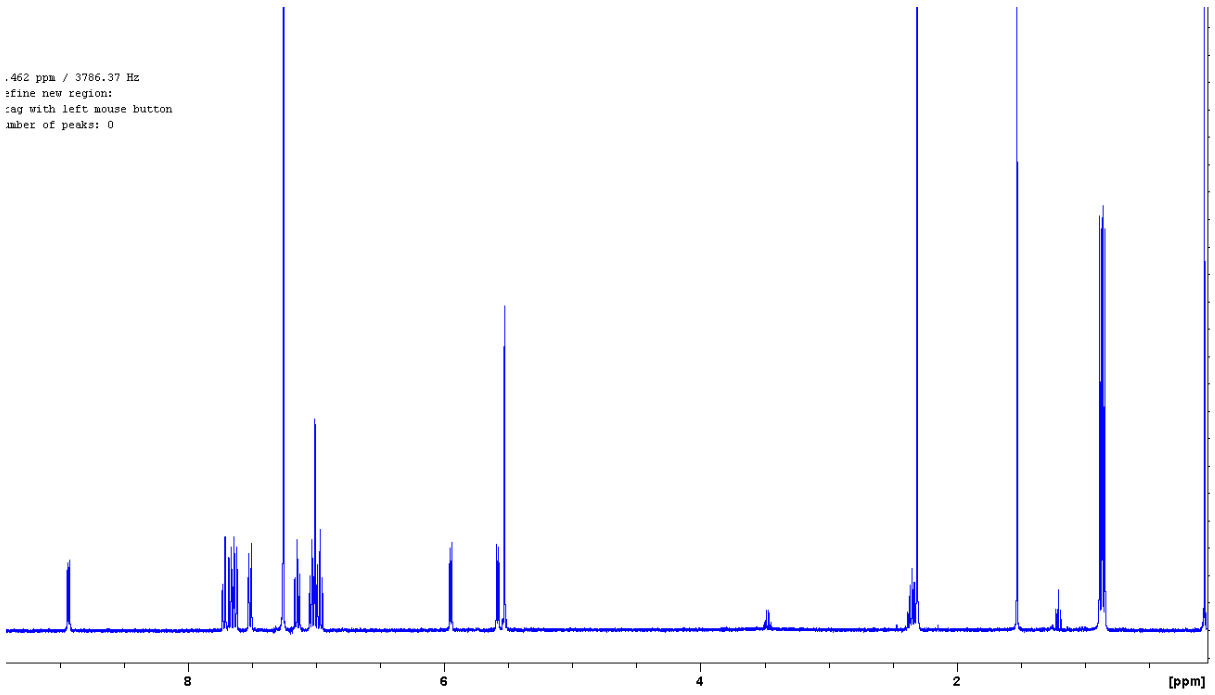

Figure S6. ^{1}H NMR spectrum of complex **3**

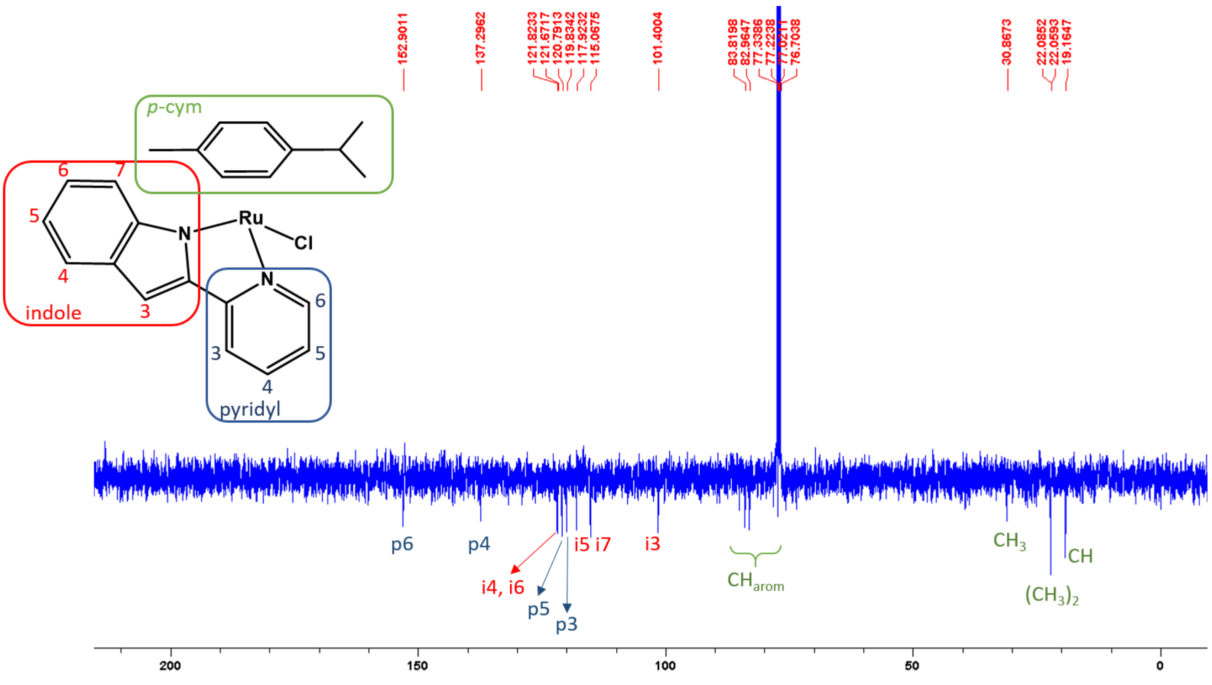

Figure S7. ^{13}C NMR spectrum of complex **3**

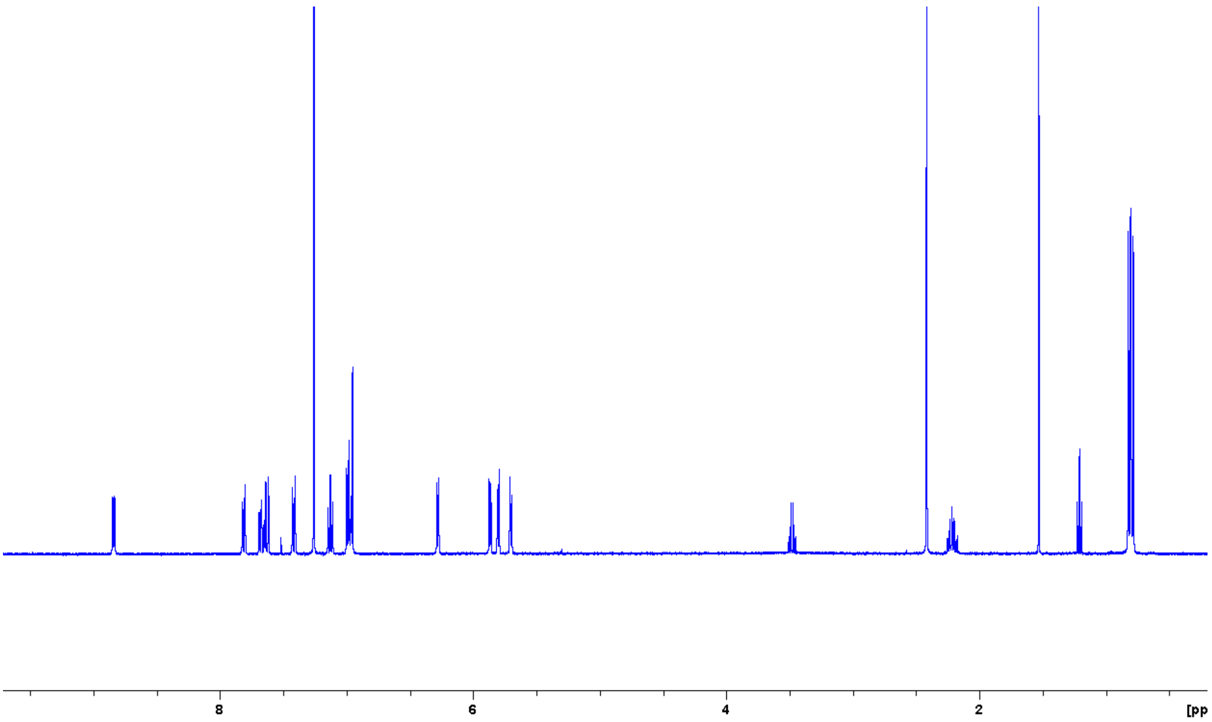

Figure S8. ^{1}H NMR spectrum of complex **4**

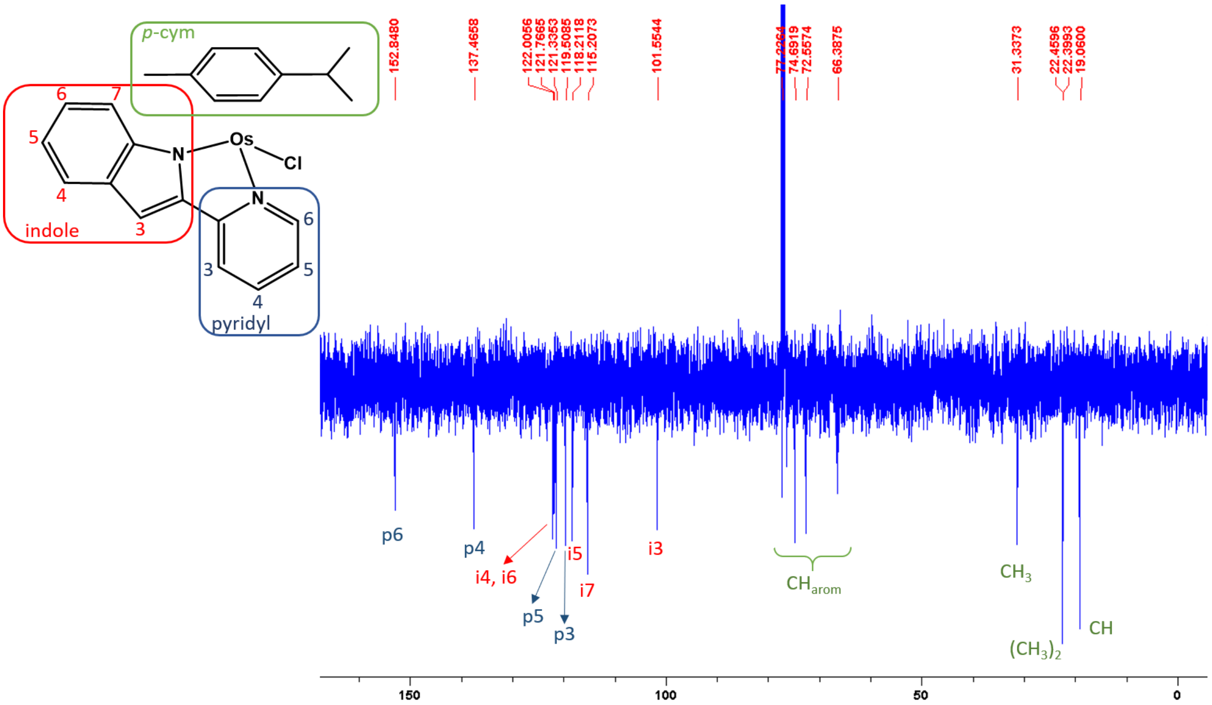

Figure S9. ^{13}C NMR spectrum of complex **4**

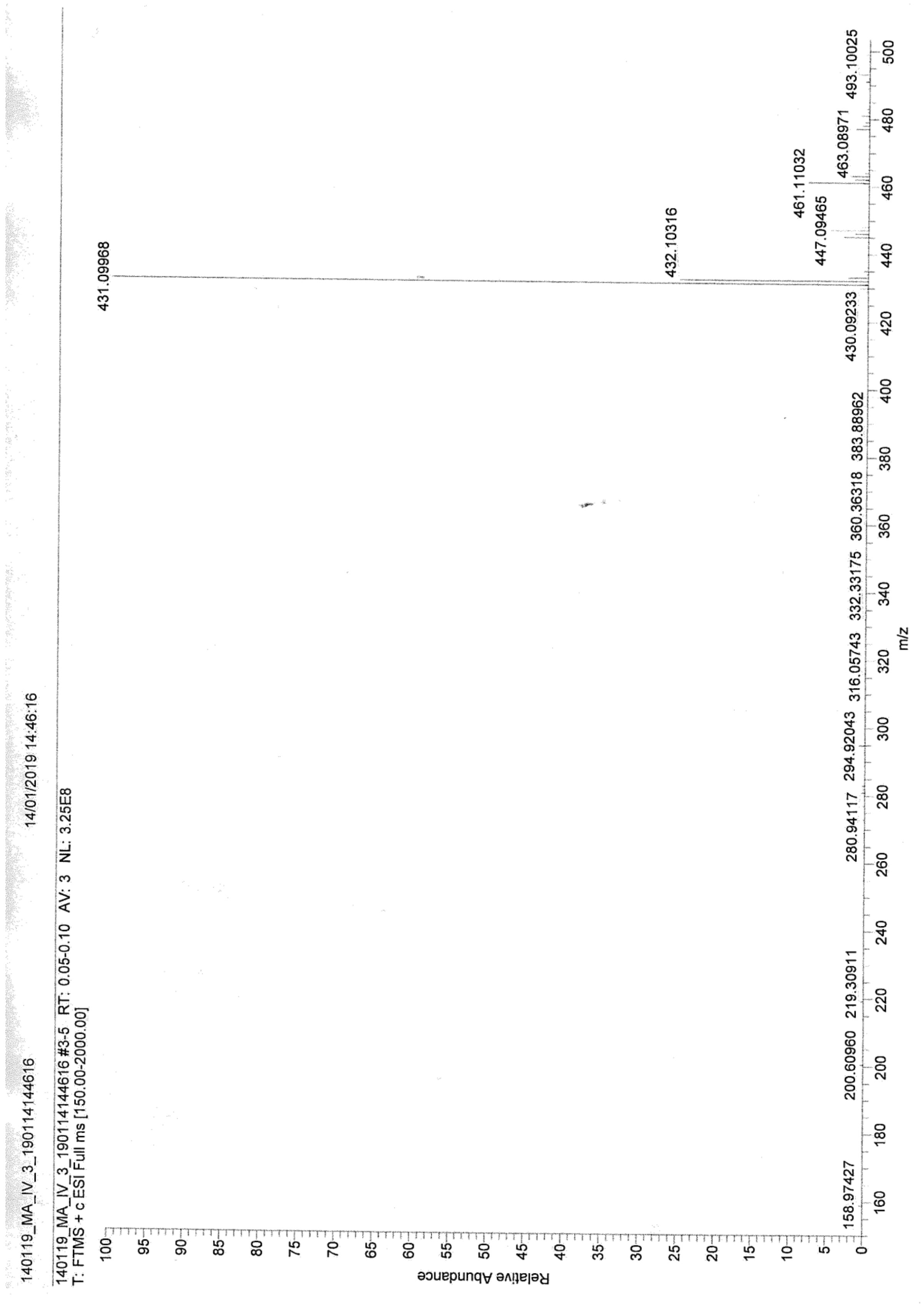

Figure S10. High-resolution ESI mass spectrum of complex **1**

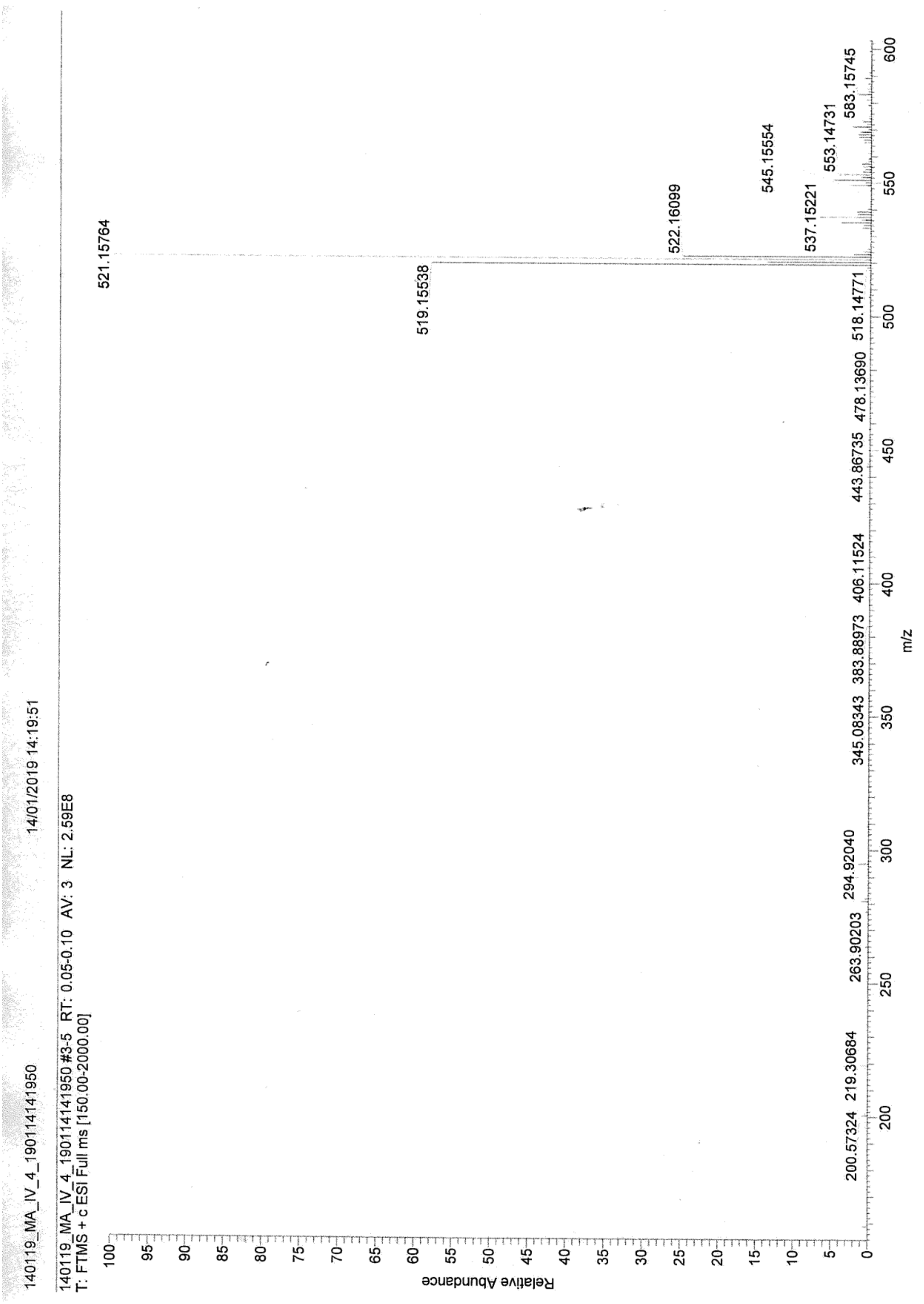

Figure S11. High-resolution ESI mass spectrum of complex **2**

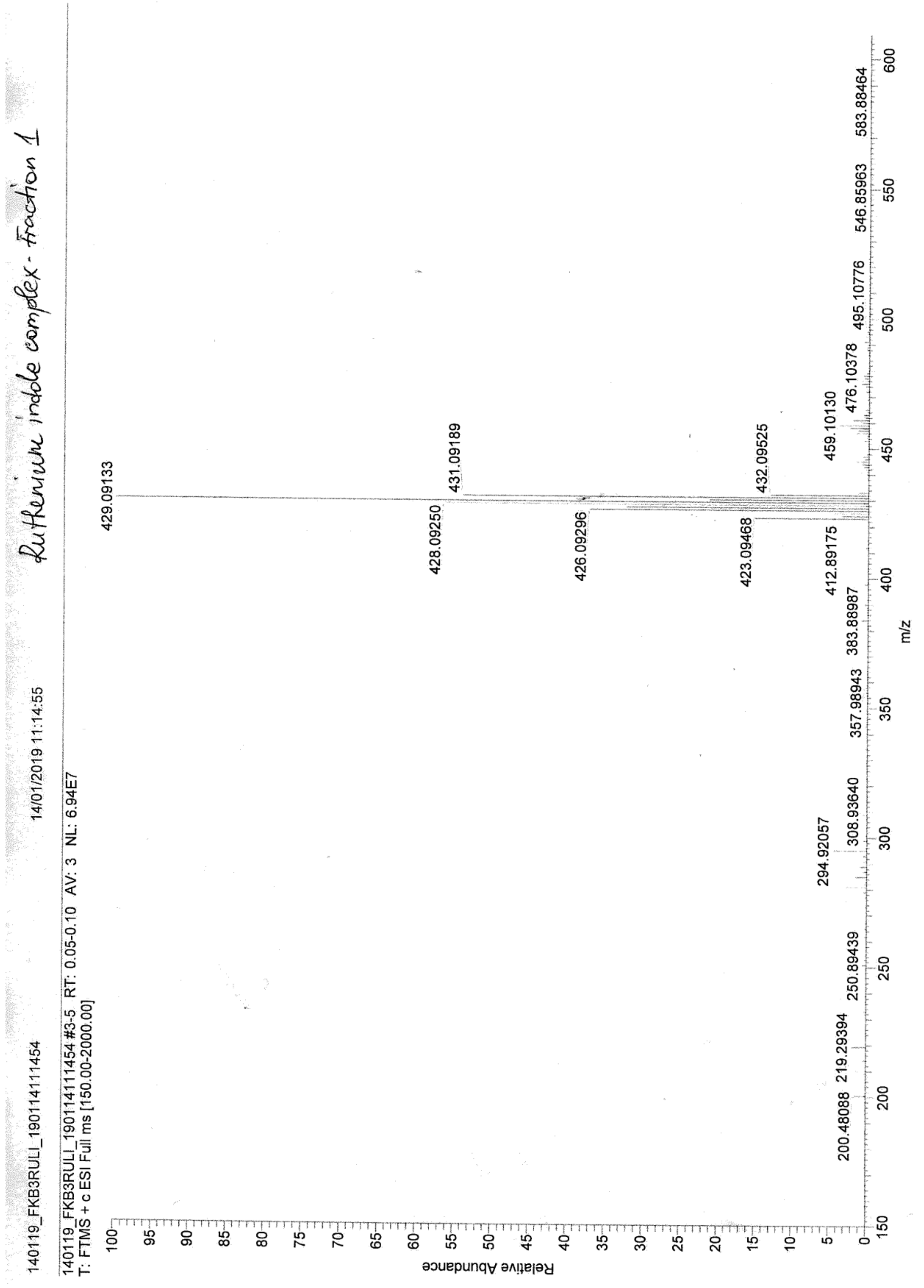

Figure S12. High-resolution ESI mass spectrum of complex **3**

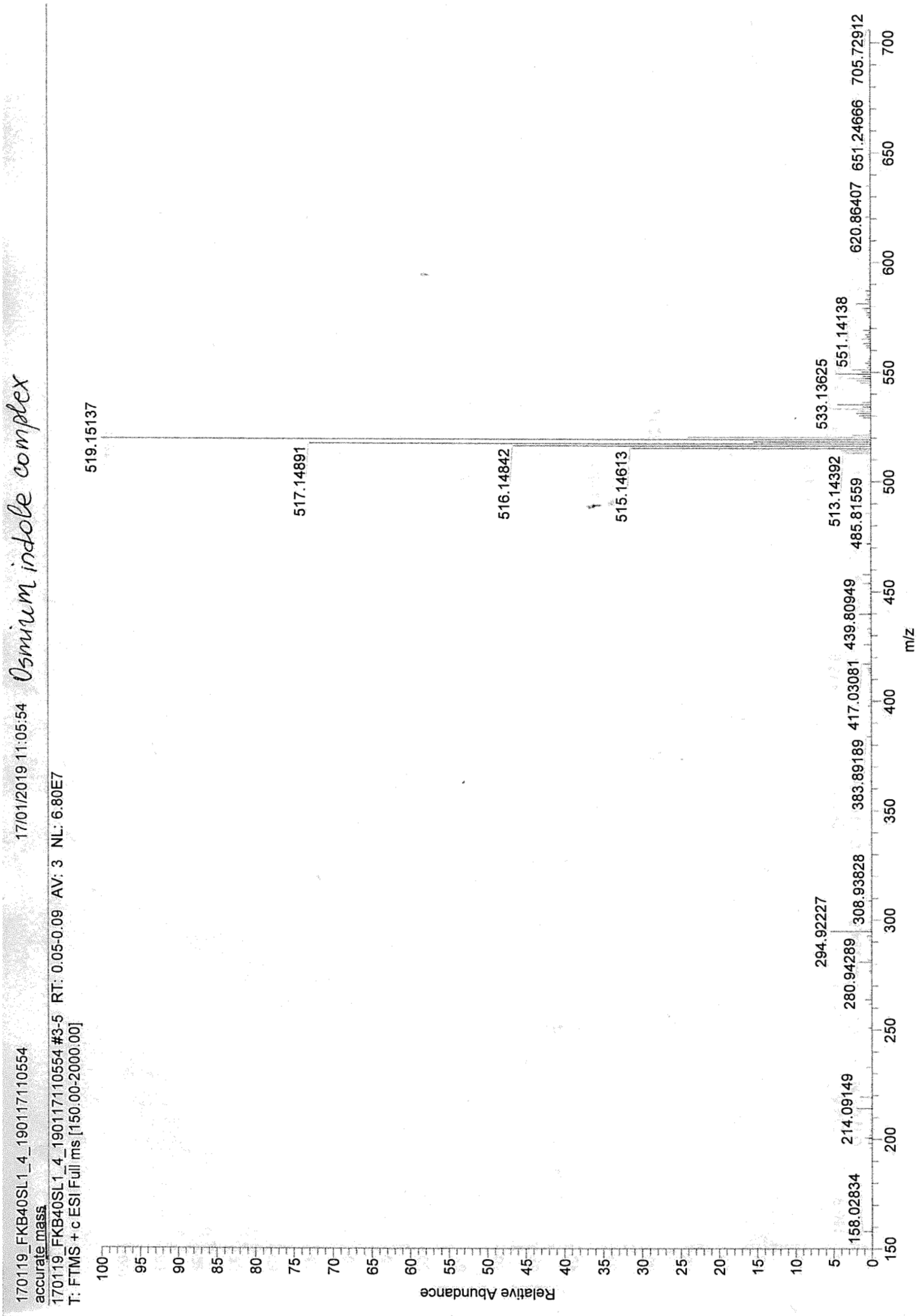

Figure S13. High-resolution ESI mass spectrum of complex **4**

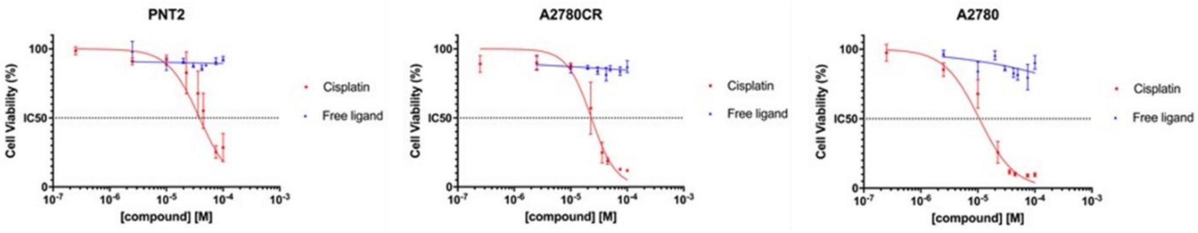

Figure S14. IC_{50} graphs for the ind-py ligand against PNT2, A2780, and A2780cisR.
